# A Similarity Regression Fusion Model for Integrating Multi-Omics Data to Identify Cancer Subtypes

**DOI:** 10.3390/genes9070314

**Published:** 2018-06-21

**Authors:** Yang Guo, Jianning Zheng, Xuequn Shang, Zhanhuai Li

**Affiliations:** School of Computer Science and Engineering, Northwestern Polytechnical University, Xi’an 710072, China; jennings@mail.nwpu.edu.cn (J.Z.); shang@nwpu.edu.cn (X.S.); lizhh@nwpu.edu.cn (Z.L.)

**Keywords:** cancer subtypes, data integration, similarity fusion

## Abstract

The identification of cancer subtypes is crucial to cancer diagnosis and treatments. A number of methods have been proposed to identify cancer subtypes by integrating multi-omics data in recent years. However, the existing methods rarely consider the biases of similarity between samples and weights of different omics data in integration. More accurate and flexible integration approaches need to be developed to comprehensively investigate cancer subtypes. In this paper, we propose a simple and flexible similarity fusion model for integrating multi-omics data to identify cancer subtypes. We consider the similarity biases between samples in each omics data and predict corrected similarities between samples using a generalized linear model. We integrate the corrected similarity information from multi-omics data according to different data-view weights. Based on the integrative similarity information, we cluster patient samples into different subtype groups. Comprehensive experiments demonstrate that the proposed approach obtains more significant results than the state-of-the-art integrative methods. In conclusion, our approach provides an effective and flexible tool to investigate subtypes in cancer by integrating multi-omics data.

## 1. Introduction

Cancer is a heterogeneous disease and usually includes several subtypes in terms of different molecule pathogeneses and clinical features [1,2,3]. It is crucial to identify cancer subtypes to improve the precision of cancer diagnosis and therapy, since different cancer subtypes may have different prognoses and treatments [4,5]. A typical example is the subtype heterogeneity in breast cancer, in which the estrogen receptor (ER)-positive breast cancer subtype responds well to hormone therapy, while the human epidermal growth factor receptor 2 (HER2)-positive breast cancer subtype responds well to chemotherapy [6]. However, we still have limited subtype knowledge for most human cancers at present.

In recent years, cost-effective genome-wide sequencing technologies have made it easier to collect diverse types of large-scale multi-omics data to study human cancers [7]. For example, The Cancer Genome Atlas (TCGA) [8,9] pilot project generated various types of genome, transcriptome, and epigenome information on over 1100 patient samples for over 34 cancer types [10]. These sequencing data provided an unprecedented opportunity for investigating cancer subtype information by capturing multi-omics data. A number of computational approaches have been proposed to identify cancer subtypes using multi-omics sequencing data in the past decade [6,7,11,12]. To identify cancer subtypes, the most common methodology is to cluster patient samples into different subtype groups by using data mining or machine learning methods on the single omics data type [13,14,15,16]. However, cancer is heterogeneous, so single omics data type may not be sufficient to sense the subtype information accurately. To account for more information, many integrative methods were proposed to integrate multiple data types to identify subtypes in cancer. The simple integrative method is to identify cancer subtypes in each individual data type, and then combine the results obtained for all data types to detect the final subtype clusters [17,18,19,20]. Nevertheless, detecting subtypes in each data type separately may lose the comprehensive information in integrative data and inconsistent results may be obtained [6,17]. Therefore, to overcome these drawbacks, many advanced approaches were developed to consider multiple data types simultaneously [21,22], such as iCluster [23,24], consensus non-negative matrix factorization (CNMF) [21,25,26], similarity network fusion (SNF) [7], etc. The iCluster is a machine learning approach that uses a joint latent variable model for integrative clustering. While it is powerful, since the high computational complexity and feature selection is necessary in practice, the clustering results largely depend on the feature selection [17]. This limits its application in extremely high-dimensional data. The CNMF is a modified non-negative matrix factorization method, which is an effective dimension reduction method to discover biological patterns from high-dimensional data by using non-negative matrix factorization [25]. One recent method, SNF [7], uses the similarity networks between samples in multi-omics data as a basis for integration. The SNF approach was demonstrated to be effective at obtaining promising results in data integration. However, on the one hand, the SNF is a complexity model, which uses iterative approach to update the similarity networks between samples, and it is hard to be interpreted in some extent. On the other hand, SNF does not consider the weight of each data-view, while different data types may provide different contributions to the clustering in data integration, since different data noise may be included in different omics data. Recently, the affinity network fusion (ANF) [27] method was proposed to upgrade the performance by fusing multi-view affinity networks according to random walk steps. Although it was proven that ANF obtained a better performance than SNF in cancer type clustering, the computation model is still complex, so a simpler and more powerful model needs to be developed. In addition, most of the existing methods consider the similarity between samples directly and the similarity biases in each data type are ignored. However, since most of the biological data are highly dimensional, the outlier features may have a large effect on the similarity calculation. Therefore, there is uncertainty when predicting the similarity by using high-dimensional features directly in a single data type. This uncertainty may introduce similarity biases in integrative similarity estimation to some extent.

Beyond considering the cancer analyses in the profiling of omics levels, other statistical methods incorporate the latent variables or factors in the model and learn the corresponding variables according to optimization. The relationships of latent variables can be defined by specific molecular characteristics of cancers. For example, the PARADIGM [28] used the pathway-level genes as the factors in the model to detect the molecularly defined cancer subgroups. Since PARADIGM used the pathway index model to depict the prognostic risk of each pathway individually, it is not able to determine the joint effects of pathways and the relative importance of each selected pathway in cancer analyses [29]. This may limit its overall performance to some extent. The Integrative Genomics Robust iDentification (InGRiD) of cancer subgroups [29] considered the overlap of pathways and integrated gene expression with biological pathways to improve the robustness of identification and interpretation of molecularly defined subgroups of cancer patients. However, InGRiD is a semi-supervised method and extensional survival information needs to be used in supervised learning. In addition, InGRiD only integrates the profile of genes at the pathway level and other omics data need to be further considered. Therefore, to improve the understanding of cancer subtypes, a more powerful and flexible approach needs to be developed to integrate multi-omics data to identify subtypes in cancer.

In this paper, we consider the similarity biases between samples and data-view weights in multi-omics data integration for cancer subtype prediction. We propose a similarity regression fusion (SRF) model to integrate multi-omics data to identify cancer subtypes. To obtain more accurate similarity estimation between samples, for each sample pair in each individual data type, we integrate the similarities between them and their neighbors in all data types, and use a generalized linear regression model to learn the data associations between samples by considering the similarity biases. Then, based on the learned model, we can more accurately predict the similarity between samples. In each data type, we predict the corrected similarity between samples by integrating the similarity information in other data types. We integrate all predicted similarities between samples in all data types according to different data-view weights. Finally, based on the integrative similarity information of samples, we use the spectral clustering approach [30] to cluster samples into different cancer subtype groups.

Comparing with the existing approaches, in this paper, the main contributions of the proposed model (SRF) can be summarized as follows: (1) The SRF model is a more simple and interpretable approach. It needs much less computation and the overall procedure of the algorithm is clearer; (2) The similarity biases between samples are considered to estimate a corrected version of similarities between samples by integrating the similarities between them and their neighbors; (3) The contribution weight of each data type is considered in a similarity fusion process. Comprehensive experiments based on four cancer type datasets demonstrate that the proposed SRF approach provides an effective and flexible tool to identify cancer subtypes by integrating multi-omics data. The source code of the tool is freely available at https://github.com/yiangcs001/SRF.

## 2. Materials and Methods

### 2.1. Datasets

In this study, we use four cancer types of data from TCGA, processed and provided by [7]. The four cancer types include breast invasive carcinoma (BIC) with 105 samples, glioblastoma multiforme (GBM) with 215 samples, lung squamous cell carcinoma (LSCC) with 106 samples, and colon adenocarcinoma (COAD) with 92 samples. For each cancer, the data include the gene expression, DNA methylation, and microRNA (miRNA) expression data. In addition, for each cancer, the corresponding clinical survival data are included. Table 1 shows the details of datasets.

### 2.2. Methods

**Figure 1:**
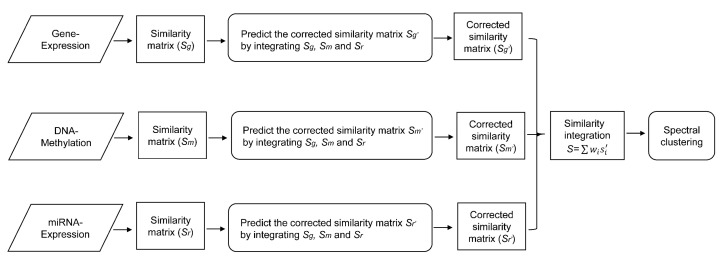
Illustration of the proposed similarity fusion approach. Suppose gene expression, DNA methylation, and miRNA expression data are integrated to identify cancer subtypes.

In this section, we introduce a proposed SRF model, which integrates multi-omics data to identify cancer subtypes. Specifically, we calculate the similarities between samples using the raw features in each dataset and predict the fusion similarities between samples in multi-omics data. We cluster all patient samples into different subtype groups by employing the fusion similarity information between samples. Figure 1 shows an illustration of the overall procedure of our integrative model.

#### 2.2.1. Correlation Similarity

Suppose K types of omics profile data are considered in analyses and there are *n* patient samples in each type of data. In each dataset $D_{k,\;n\times m}$, where *n* and *m* are the number patients and omics features in dataset k, respectively. $x_{k,i}\in D_{k,\;n\times m}$ is the vector of sample *i* in *m* features in dataset k. We calculate the Pearson correlation-based similarities between patient samples based on the corresponding profile data. Specifically, the correlation coefficient (similarity) between patient i and j in dataset k can be defined by

(1) $$\;S_{k,ij}=\frac{\sum_{t=1}^{m}\left(x_{k,it}-{\bar{x}}_{k,i}\right)\left(x_{k,jt}-{\bar{x}}_{k,j}\right)}{\sqrt{\sum_{t=1}^{m}{\left(x_{k,it}-{\bar{x}}_{k,i}\right)}^{2}}\sqrt{\sum_{t=1}^{m}{\left(x_{k,jt}-{\bar{x}}_{k,j}\right)}^{2}}}$$

where $x_{k,it}$ and $x_{k,jt}$ are the values in feature t for patient samples i and j in dataset k. ${\bar{x}}_{k,i}$ and ${\bar{x}}_{k,j}$ are the average values of $x_{k,i}$ and $x_{k,j}$.

In each dataset k, we calculate the correlation similarity matrix $M_{k}$ between patients based on the profile data, where $M_{k,ij}$ is the Pearson correlation coefficient between patient i and j in dataset k. For all K datasets, the correlation similarity matrix set $M=\left(M_{1},\;M_{2},\ldots ,M_{K}\right)$.

#### 2.2.2. Similarity Regression Fusion

An accurate similarity measurement is important to predict more accurate subtype groups. In this section, we introduce a novel similarity fusion method that integrates all similarity associations in multi-omics datasets to predict a fusion similarity matrix, which has higher confidence to depict the similarities between patient samples.

Given the correlation similarity matrix set $M=\left(M_{1},\;M_{2},\ldots ,M_{K}\right)$ in K datasets. For each $M_{k,ij}\in M_{k}$, the Fisher transformation [31,32] form $r_{k,ij}$ is employed to measure the correlation similarity in real space.

(2) $$r_{k,ij}=r_{k,ji}=\frac{1}{2}ln\left(\frac{1+M_{k,ij}}{1-M_{k,ij}}\right)$$

We transform all correlation similarity matrices $M=\left(M_{1},\;M_{2},\ldots ,M_{K}\right)$ to $M^{\prime }=\left(M_{1}^{\prime },\;M_{2}^{\prime },\ldots ,M_{K}^{\prime }\right)$ using Equation (2), and then predict accurate similarities between patients by using the regression fusion method.

For each pair of patient i and j in dataset k, we assume $p_{k,ij}$ is the possibility of i and j in the same subtype supported by dataset k. We use a generalized linear regression model to estimate $p_{k,ij}$ by integrating the similarity information between them in all other types of omics datasets. Specifically, given the Fisher transformed similarity matrix set $M^{\prime }=\left(M_{1}^{\prime },\;M_{2}^{\prime },\ldots ,M_{K}^{\prime }\right)$ in K datasets, $\forall r_{k,ij}\in M_{k}^{\prime }$, we assume

(3) $$logit\left(p_{k,ij}\right)=\mu \cdot r_{k,ij}^{\prime }$$

(4) $$r_{k,ij}^{\prime }\;\sim \;N\left(\beta_{0}+\sum_{t\neq k}\beta_{t}r_{t,ij},\sigma_{k,ij}^{2}\right)$$

(5) $$p_{k,ij}=\frac{e^{\mu r_{k,ij}^{\prime }}}{1+e^{\mu r_{k,ij}^{\prime }}},\;k=1,2,\ldots ,K$$

where $r_{k,ij}^{\prime }$ is the corrected similarity between i and j in dataset k, which is predicted by using the similarities between i and j in all other datasets except k. $\mu \in R^{+}$ is a hyper-parameter that can be empirically set in practice to scale the predicted data range; the default is 3.0 in this study. The Gaussian distribution models the estimation uncertainty of the similarity $r_{k,ij}^{\prime }$ between i and j in dataset k. $r_{k,ij}^{\prime }$ can be predicted by using the learned regression model. $\beta_{t}$ is the parameter in the linear model, and the $\sigma_{k,ij}^{2}$ is the variance (bias) of similarity between i and j in dataset k.

#### 2.2.3. Parameter Learning

The parameters of the generalized linear regression model can be estimated by using maximum likelihood estimation (MLE) or latest square method. Given the transformed similarity matrix set $M^{\prime }=\left(M_{1}^{\prime },\;M_{2}^{\prime },\ldots ,M_{K}^{\prime }\right)$ in all types of omics datasets, for each pair of patients i and j, $r_{k,ij}\in M_{k}^{\prime }$, we estimate a corrected form $r_{k,ij}^{\prime }$ by considering the bias of it in each omics dataset. To predict the corrected form $r_{k,ij}^{\prime }$ by using the linear regression model, we need to learn all the parameters in the model first.

For the transformed similarity between i and j in dataset k, $r_{k,ij}\in M_{k}^{\prime }$, we train a generalized linear model using the similarities between them and other samples, and then we use the learned model to predict the corrected form $r_{k,ij}^{\prime }$ by using the similarities between i and j in other datasets. Specifically, for the model of $r_{k,ij}\in M_{k}^{\prime }$, the training data is

(6) $$T{\left(X,Y\right)}_{r_{k,ij}}=\{\begin{array}{l} X=\left\{vec_{p}\left(r_{p,it}\cup r_{p,jt}\right)\right\},\;\;\;\;where\;p=1,2,\ldots ,K;\;\;p\neq k,\;\;t\neq i,j;\\ Y=vec_{k}\left(r_{k,it}\cup r_{k,jt}\right)\;,\;\;\;\;\;\;\;where\;t\neq i,j; \end{array}$$

where $vec_{p}\left(r_{p,it}\cup r_{p,jt}\right)$ is the union vector of the similarities between i and its other neighbors, and between j and its other neighbors in dataset p; $vec_{k}\left(r_{k,it}\cup r_{k,jt}\right)$ is the union vector of the similarities between i and its other neighbors, and between j and its other neighbors in dataset k.

In each dataset, we correct the corresponding similarity matrix between patients by employing the similarity matrices in other omics datasets. For each pair of patients, we use the similarities between them and their neighbors to train a generalized linear regression model, and thus use the learned model to predict the corrected similarity between them based on their similarities in all other types of omics datasets. In this study, we use the complete similarity network between patients as basis. We use this training strategy based on two assumptions: (1) If two patients have a similar cancer subtype attribution in one omics dataset, they also tend to have similar cancer subtype attribution in other related omics datasets; (2) If two patients have a similar cancer subtype attribution in one omics dataset, they also tend to have similar correlation distribution to their neighbors in the same omics data. These two principles are consistent with the fact that patients with a similar cancer subtype should have a similar correlation distribution to their neighbors, not only in the one specific omics dataset but also in the other related omics datasets. We train a generalized multivariable linear regression fusion model for each pair of patients in each dataset and predict a corrected version of similarity between them, which integrates the similarity information between them in multi-omics datasets. In practice, the regression model can be learned in integrative or separate form. In this paper, since the sample size is relatively small in each cancer type dataset, we use the integrative learned form by concatenating the similarities in multi-omics data together and thus learn a unified model in all types of omics data. In general, if the data sample size is large or the data type is more than three, we can learn a separate model in each dataset.

#### 2.2.4. Similarity Integration and Cancer Subtype Prediction

Based on the learned generalized linear regression model, we can predict the possibilities of patient pairs belonging to the same cancer subtype in each dataset by using Equations (3)–(5). This possibility provides more accurate similarity information between patients. Indeed, to obtain more discriminated similarity measurements, we use a scaled exponential monotone function to determine the final similarity between patients in each dataset. Given $PM=\left(PM_{1},\;PM_{2},\;\ldots ,PM_{K}\right)$ are the similarity possibility matrices in all omics datasets. $p_{k,ij}\in PM_{k}$ is the possibility of the patient i and j belonging to the same subtype predicted in dataset k. The scaled $p_{k,ij}^{\prime }\in PM_{k}{}^{\prime }$ is defined as

(7) $$p_{k,ij}^{\prime }=\frac{1}{1+e^{p_{k,ij}^{-t}}}$$

where $t\in R^{+}$ is a hyper-parameter that can be set by the user, and the default value in this study is 1.0.

Accounting for the scaled similarity matrices $PM^{\prime }=\left(PM_{1}{}^{\prime },\;PM_{2}{}^{\prime },\;\ldots ,PM_{K}{}^{\prime }\right)$ in all datasets, we incorporate all of them by using different weights in different data views to predict the final integrative similarity matrix between patients. The final integrative similarity matrix between patients is defined as

(8) $$W=\sum_{k=1}^{K}w_{k}\cdot PM_{k}^{\prime },\;\;s.t.\;\sum_{k=1}^{K}w_{k}=1$$

where $w_{k}$ is the weight of the scaled similarity matrix predicted in dataset k, which can be defined by the user in a flexible manner.

With the predicted integrative similarity matrix W, we use the spectral clustering method to identify cancer subtype clusters. Concretely, we want to identify m subtype clusters in n samples. A cluster partition matrix $Y^{m\times n}=\left(y_{1},y_{2},\ldots ,y_{n}\right)$ is used to indicate the labels of samples. For each sample $x_{i}$, the cluster indicator vector $y_{i}\in {\left\{0,1\right\}}^{m}$, where $y_{i}\left(k\right)=1$ if sample $x_{i}$ belongs to the $k^{th}$ cluster, otherwise $y_{i}\left(k\right)=0$. The spectral clustering method identifies the cluster labels by solving an optimization problem [7,27]:

(9) $$\underset{Q\in R^{n\times m}}{\min}Trace\left(Q^{T}L^{+}Q\right),\;\;s.t.\;Q^{T}Q=I$$

where $Q=Y{\left(Y^{T}Y\right)}^{-1/2}$ is the function of partition matrix Y, $L^{+}=I-D^{-1/2}WD^{-1/2}$ is the normalized Laplacian matrix given the similarity matrix W. D is the diagonal matrix, with diagonal elements being the sum of each row in W.

## 3. Results

### 3.1. Parameter Selection

In our model, the only hyper-parameter that needs to be tuned is the integration weight $w=\left(w_{1},\;w_{2},\;\ldots ,w_{K}\right)$, which defines the weight of each omics data type in integration. Specifically, in our study, three omics data types are integrated according to $w=\left(w_{1},\;w_{2},w_{3}\right)$, which defines the data weight of the predicted similarity between samples in gene expression ($w_{1}$), DNA methylation ($w_{2}$) and miRNA expression ($w_{3}$), respectively (Equation (8)). Different similarity weights may lead to different cancer subtype predictions. To select a better weight combination in each cancer type data, we searched all three-dimensional weight combination spaces with a 0.05 differential each time, and evaluated the performance of each model in all omics data types. As studied in [7], we had five subtype groups in BIC, three subtype groups in GBM, four cancer subtype groups in LSCC, and three cancer subtype groups in COAD. In this study, to be consistent with the previous study and provide for fair comparisons, we set the same expected cluster number in our downstream analyses to identify different cancer subtypes in each cancer dataset. For the identified cancer subtypes, we performed survival significance analyses in patients and evaluated the Cox log-rank test [33] *p*-value performance using different weight combinations.

**Figure 2:**
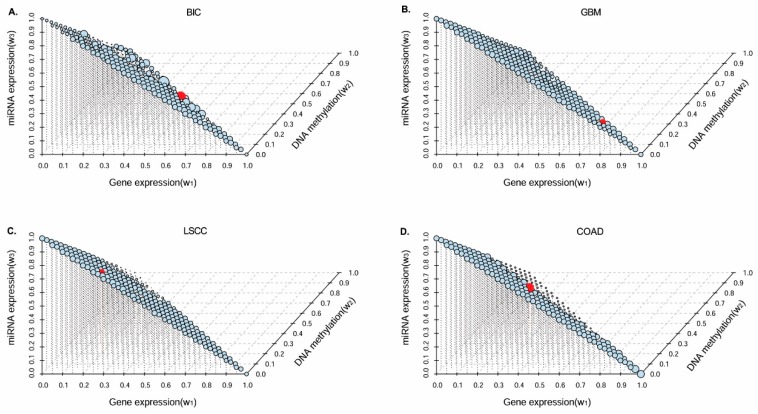
The Cox log-rank test *p*-value distribution in survival analyses of similarity regression fusion (SRF) identified subtypes by using different weight parameters in integration. Gene expression, DNA methylation, and miRNA expression data were used in this study. The size of dot was determined by −log(*p*-value). We selected weight parameters with the smallest *p*-value in each cancer data (red dot) in our study. (**A**–**D**) Cox log-rank test *p*-value distribution using differently integrative weights in BIC, GBM, LSCC, and COAD cancer datasets, respectively.

Figure 2A–D shows the Cox log-rank test *p*-value performance in survival analyses for the identified cancer subtypes by using our approach for all possible weight combination searching spaces (0.05 step-length) in the four cancer datasets. In each plot, the size of each dot corresponds to the negative logarithm *p*-value in survival analyses. As shown in Figure 2A, our method obtains significant *p*-values in survival analyses in most weight combinations to integrate the three omics data types in BIC cancer datasets. This demonstrates that our approach is generalizable and robust to different parameter configurations. Especially when the combination weight *w* = (0.45, 0.5, 0.05) for gene expression, DNA methylation, and miRNA expression similarity matrices (red dot), respectively, we obtain the most significant subtype estimation in survival analyses (*p* = 1.20 × 10^−6^). In Figure 2B, we obtain a significant *p*-value in survival analyses for most of the weight combinations in GBM cancer datasets, and the most significant *p*-value (*p* = 2.66 × 10^−4^) was obtained when *w* = (0.7, 0.25, 0.05), corresponded to the predicted similarity matrices from the gene expression, DNA methylation, and miRNA expression datasets, respectively. We find a similar prediction performance in all possible weight combinations in LSCC (Figure 2C) and COAD (Figure 2D) cancer datasets.

Specifically, as shown in Figure 2C, we obtain the most significant cancer subtype estimation with integrative weight *w* = (0.2, 0.2, 0.6) (*p* = 1.98 × 10^−3^) in LSCC cancer datasets; in Figure 2D, we obtain the most significant cancer subtype estimation with integrative weight *w* = (0.25, 0.45, 0.3) (*p* = 1.96 × 10^−3^) in COAD cancer datasets. Based on the performance investigations using different weight combinations for all types of omics data, we selected the best weight combination parameters to identify the cancer subtypes in each cancer type data in our downstream analyses. In addition, we notice that there are different contributions of different types of omics data to the identification of significant cancer subtypes. For example, in BIC and GBM cancer datasets, the similarity matrices in gene expression and DNA methylation datasets make more important contributions than in the miRNA expression dataset. However, in LSCC cancer datasets, the similarity matrix in the miRNA expression dataset tends to make more of a contribution than in gene expression and DNA methylation datasets; in COAD cancer datasets, the three types of omics data make roughly similar contributions to subtype prediction.

### 3.2. Performance Evaluation in Various Cancer Datasets

We first compared the integrative model with the models using only a single type of the corrected similarity information across the four cancer types. We obtained the cancer subtypes in each individual omics dataset by using the corrected similarity matrix and clustering patients into different subtype groups using spectral clustering. In particular, each individual similarity matrix between patients is predicted by using multi-omics data, but using different omics data types as evidence (as described in Section 2.2.2). For the performance comparison, we introduce two commonly used measures: (1) the *p*-value of Cox log-rank test in clinical survival analysis to evaluate the survival significance of the identified cancer subtypes; (2) a silhouette score to measure the coherence of the identified cancer subtype clusters. Table 2 shows that the integrative model obtains more significant *p*-values in survival analyses than the similarity information supported in single omics data, and all of the identified cancer subtype clusters are coherent (silhouette score ≥ 0.15) in all four cancer types.

To evaluate the overall performance of SRF in cancer subtype identification, we compared SRF with six other commonly used integrative methods, including CNMF [21,26], iCluster [23], consensus clustering (CC) [22], SNF [7], SNF-CC [21] and ANF [27]. Consensus non-negative matrix factorization uses a non-negative matrix factorization method to integrate multiple sources of data to discover cancer patterns. iCluster is a machine learning approach that uses a joint latent variable model for integrative clustering. CC is a method to define the consensus clusters in single or multiple types of omics data. SNF is a multiple omics data fusion method based on similarity networks. Similarity network fusion- consensus clustering integrates both SNF and CC: it first applies the SNF algorithm to calculate patient similarity matrices, and then uses the CC algorithm to discover subtype groups based on the similarity matrices. Affinity network fusion is a method that fuses multi-view affinity networks according to random walk to identify cancer subtypes. We performed cancer subtype clustering by using different methods in each of the four cancer datasets, and we evaluated the Cox log-rank test *p*-value in survival analyses for the identified cancer subtypes. Due to the high computational complexity of iCluster, feature selection is necessary to integrate high-dimensional data in practice. In our experiments, to run the iCluster method in all datasets, if the number of features in one dataset is over 10,000, we select 10% of the features as the data representation; otherwise, we use all features. In detail, since the datasets we used are normalized, all features actually have same variances in samples. It is impossible to select the important features using the large variance standard in samples. To test the overall performance of iCluster, we ran iCluster five times by randomly selected 10% of the features in high-dimensional datasets in experiments, and used the median of survival *p*-values as the final reference. Table 3 shows the comparison performance of each method in survival analyses in all cancer types. SRF obtains more significant results than the alternative methods in all experimental cancer types. In the LSCC and COAD cancer data, although iCluster obtains a similar *p*-value performance, SRF still shows slightly better clinical subtype estimation. This may be because the different feature selections may obtain different estimation performance for iCluster. However, according to the experiments in [7], iCluster did not obtain estimations as good as SNF using more features in most of the cancer datasets. In conclusion, this illustrates that SRF is an effective method to identify cancer subtypes by integrating multiple types of omics data. Actually, the SNF model also considers the similarity associations between patients in different types of data; however, our SRF considers not only the similarity associations between patients supported by different types of data, but also the weight of each data view in integration. This may be one important reason that SRF has better performance than SNF in data integration.

### 3.3. Cancer Subtype Clustering in Breast Cancer Data

**Figure 3:**
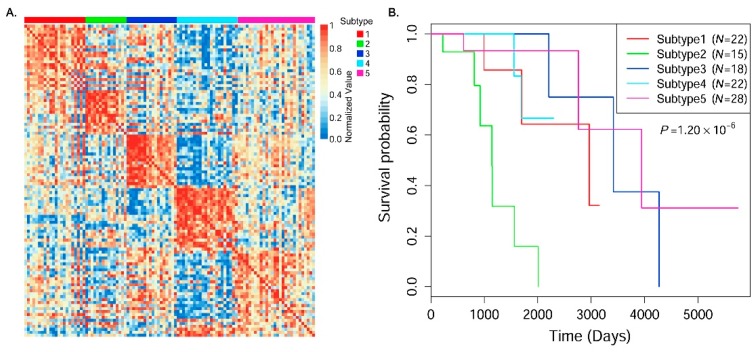
The identified subtypes in BIC cancer datasets. (**A**) Heatmap of the predicted integrative similarity matrix between samples (row normalized, arranging samples by predicted subtype labels); (**B**) Kaplan–Meier survival probability curves of patients in the identified subtype clusters. The Cox log-rank test *p*-value was included.

We used SRF to identify cancer subtypes in breast cancer data and evaluated the identified cancer subtype clusters based on several types of clinical data. With three different types of omics profile datasets, SRF predicted the integrative similarity matrix between patients and identified five cancer subtypes in breast cancer. The five cancer subtypes in our study were defined as in a previous study [7]. To investigate the consistency of patients in each subtype cluster and the differences across the identified subtype clusters, we generated a heatmap of the estimated integrative similarity matrix between patients by arranging samples according to the predicted cluster labels (Figure 3A). There are very clear block boundaries for all identified cancer subtype clusters. To examine the discrimination of survival time in the identified subtype clusters, Kaplan–Meier survival analysis [34], commonly used in survival function analysis, was performed in subtype clusters, and the Cox log-rank test *p*-value was used to measure the significance of survival difference in cancer subtypes. As shown in Figure 3B, there are very different survival patterns among different subtype clusters, and the log-rank test *p*-value is very significant (1.20 × 10^−6^). This demonstrates that SRF can identify survival meaningful subtype clusters by integrating multi-omics data in breast cancer.

We also investigated the average initial diagnosis age and survival time (only used status = 1 samples) of patients in each individual subtype cluster. As shown in Table 4, there are different average age values (initial diagnosis) and survival times in the identified cancer subtypes, especially between subtype clusters 2 and 3, in which the difference in the average survival time is more than 2-fold, and the difference in the average initial diagnosis age is more than six years. This may illustrate that there are very different pathogeneses in the two subtypes.

Five cancer subtypes in breast cancer had been reported based on the PAM50 [35] standard in TCGA, which was defined by analyzing gene expression of a few biomarkers. We analyzed the overlaps of the identified subtype clusters with the PAM50 subtypes. The five PAM50 subtypes include 23 Basal, 11 Her2, 55 LumA, 12 LumB and two Normal in the BIC data. We examined the difference between the identified subtypes and the PAM50 subtypes. As shown in Table 5, there are major overlaps of the identified subtypes with PAM50, although some differences exist. For example, most of the Basal and Her2 patients in PAM50 are enriched in clusters 4 and 5, respectively. Meanwhile, most of the LumA patients in PAM50 are enriched in clusters 1, 3, and 5, but the LumB patients in PAM50 are enriched in clusters 1 and 2. This illustrates that the identified subtypes are roughly consistent with the PAM50 subtypes (adjusted rand index (ARI) = 0.20), although there are some differences between them. This may be because the PAM50 subtypes are only based on a profile analysis of a few gene biomarkers, while our results are based on all genomic features in three omics datasets, which tends to provide more clinically meaningful subtype prediction. In addition, we investigated the ER positive/negative and progesterone receptor (PR) positive/negative clinical characteristics in the identified subtypes (omitting samples with no related information). We found that most of the patients in clusters 1, 2, 3, and 5 have both an ER+ and PR+ clinical diagnosis. Especially in cluster 3 (average survival time: 3299.3 days), all 18 patients have both an ER+ and PR+ diagnosis. This is consistent with the clinical observation that patients with ER+ and/or PR+ have a favorable prognosis and better survival outcome [36]. In conclusion, SRF identified clinically meaningful subtypes by integrating multi-omics data in breast cancer.

### 3.4. Cancer Subtype Clustering in Glioblastoma Multiforme Data

**Figure 4:**
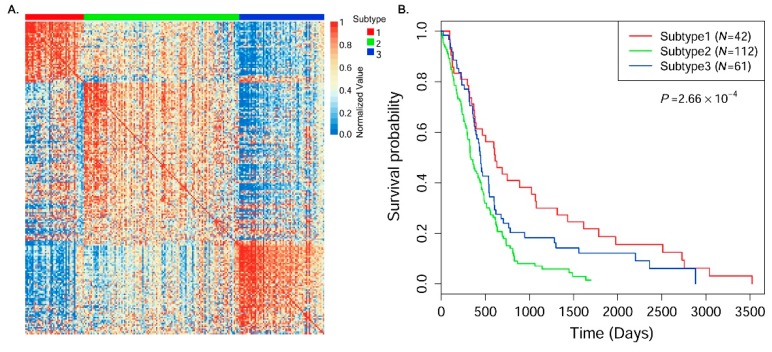
The identified subtypes in GBM cancer datasets. (**A**) Heatmap of the predicted integrative similarity matrix between samples (row normalized, arranging samples by predicted subtype labels); (**B**) Kaplan–Meier survival probability curves of patients in the identified subtype clusters. The Cox log-rank test *p*-value was included.

We used SRF to identify cancer subtypes in GBM cancer data. Consistent with a previous study [7], three subtypes were defined in our study. Figure 4A shows the heatmap of the estimated integrative similarity matrix between patients by arranging samples according to the predicted cluster labels. There are very clear block boundaries for all identified cancer subtype clusters. Figure 4B shows the Kaplan–Meier curves of all identified subtypes in survival analyses. There are significantly different survival patterns in the subtype clusters (*p* = 2.66 × 10^−4^). This suggests that the identified subtypes are clinically meaningful in terms of survival time estimation. Similarly, we investigated the average initial diagnosis age and survival time (only used status = 1 samples) of patients in each individual subtype cluster.

As shown in Table 6, there are different average age values (initial diagnosis) and survival times in the identified cancer subtypes, especially between subtype clusters 1 and 2, in which the average survival time between cluster 1 and cluster 2 is more than doubled, and the difference of the average initial diagnosis age is more than 12 years. This may illustrate that there are very different pathogeneses in the two subtypes. Interestingly, we notice that, in cluster 1, the average initial diagnosis age is relatively small (46.4 years), although the average survival time is the longest (931.9 days). This suggests that the occurrence of this subtype may have age-specific characteristics.

According to the TCGA clinical data, four clinical subtypes were reported based on gene expression in GBM. We investigated the overlaps of the identified subtype clusters with the annotated subtypes. The four annotated subtypes include 58 Classical, 66 Mesenchymal, 34 Neural, and 57 Proneural in GBM data. Table 7 shows the overlaps between the identified subtypes and annotated subtypes. We see that most patients with Classical, Neural, and Proneural labels are enriched in cluster 2, 2, and 1, respectively. The patients with Mesenchymal labels are enriched in both clusters 2 and 3. This demonstrates that the identified subtypes provide consistent clinical subtype information to some extent (ARI = 0.173).

**Figure 5:**
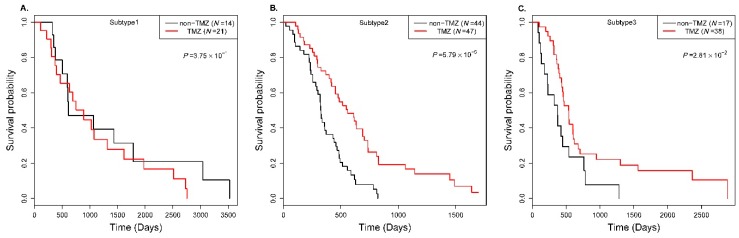
Kaplan–Meier survival possibility curves of patients for temozolomide treatment in the identified subtype clusters in GBM cancer datasets. The Cox log-rank test *p*-value was included.

In addition, to further investigate the differential biological consequences of the identified subtypes, we researched how the patients in each individual subtype respond to treatment. Of the 215 patients in GBM data, 106 patients were treated with temozolomide (TMZ), an alkylating agent that leads to thymine mispairing during DNA replication [37], and 75 patients were not treated (non-TMZ). We investigated the survival differential of the patients with treatment versus without treatment in each individual subtype cluster. Figure 5 shows the Kaplan–Meier curves of the treatment group versus non-treatment group in each individual identified subtype cluster.

As shown in Figure 5A–C, we can see that temozolomide treatment is only effective in subtype clusters 2 and 3, which show a significant differential of survival possibility between the two groups (*p* = 5.79 × 10^−5^, 2.81 × 10^−2^). However, in subtype cluster 1, there is not a significant survival differential between the two groups (*p* = 0.375). This may illustrate that there are different biological regulations or consequences arising from glioblastoma multiforme cancer and that temozolomide may only have an effect on some specific biological regulation processes.

To further investigate the differential of the biological regulation process in the identified subtype clusters, we determined the differentially expressed genes between different subtype clusters by using the Kruskal–Wallis rank sum test [17] (BH-FDR < 1.0 × 10^−5^), and performed gene enrichment analysis of differentially expressed genes in the gene ontology [38] biological process category using DAVID tool [39]. We found that the most over-expressed genes were related to inflammatory response, extracellular organization, cell adhesion functional terms, etc. The most of under-expressed genes were linked to transcription-related functions, such as transcription and DNA-templated, negative regulation of transcription, regulation of the cell cycle, etc. (Table S1). We also saw similar enrichment patterns for the genes expressed differentially between subtypes 1 and 3 (Table S2). These results are similar to those found in [17,40] for the positive subtype. This suggests that the cancer subtypes identified by our method were more biologically meaningful.

## 4. Discussion and Conclusions

The identification of cancer subtypes is of great importance to precision medicine and personalized cancer treatment. The advance in high-throughput technologies has provided various types of biological omics sequencing and network data [41]. Several computational methods have been proposed to integrate multi-omics data to identify cancer subtypes in recent years [7,21,22,24]. The similarity network fusion method (SNF), one currently popular method, is an effective and efficient integrative method that considers the similarity network information in each type of dataset and predicts the subtype clusters by an iterative similarity network fusion. Compared with other existing methods, SNF is more effective at identifying cancer subtypes with clinical survival patterns. However, SNF is a complex model that uses an iterative approach to calculate the fusion similarity, and it is hard to interpret in practice. Moreover, SNF does not consider the weights of different omics data views, while different omics data may provide different contributions to data integration since different data noise is included. The recent ANF method upgrades the identifying performance by using multi-view affinity networks according to random walk, while the computation model is still complex, and its performance needs to be further improved. In addition, most existing methods do not consider the bias of similarity between samples in each individual omics dataset. The similarity bias may introduce errors in similarity fusion. Therefore, a more powerful and flexible approach needs to be developed to integrate multi-omics data to identify subtypes in cancer.

In this paper, to ease the abovementioned issues, we proposed a SRF model to integrate multi-omics data to identify cancer subtypes. We used a generalized multi-variable linear regression model to learn the associations of similarities between samples in all types of omics data, and then predicted the corrected version of similarity for each patient pair in each omics dataset. Finally, we integrated all corrected similarity information between samples according to different data view weights, and used the spectral clustering approach to cluster samples into different cancer subtype groups. Systematic experiments integrating gene expression, DNA methylation, and miRNA expression data in four cancer types (BIC (Figure 3), GBM (Figure 4), LSCC (Figure S1) and COAD (Figure S2)) demonstrated that the proposed SRF obtained more significant clinical subtype clusters than the existing methods. The results suggested that the proposed model of similarity regression fusion more accurately predicted sample similarities in data integration. This is important for clustering patients and identifying cancer subtypes.

By using both the SRF and SNF methods on the TCGA BIC, GBM, LSCC, and COAD datasets to integrate three types of cancer omics data (gene expression, DNA methylation, and miRNA expression) to identify cancer subtypes, we found there are highly consistent subtype predictions between their results. The adjusted rand indices (ARIs) are 0.26, 0.30, 0.40, and 0.60 on the BIC, GBM, LSCC, and COAD datasets, respectively. This suggests that SRF and SNF identified roughly similar subtypes; however, SRF sensed more accurate similarity information between samples and predicted more clinically meaningful subtype patterns according to patient survival functional analyses in subtypes (Table 3). Furthermore, by analyzing the identified cancer subtypes in GBM datasets, we found that two-thirds of subtypes responded to temozolomide treatment and there was a significant survival differential between the treatment and non-treatment groups in those two subtypes; in the other subtype, there was not a significant survival differential between the treatment and non-treatment groups. The results suggest that different molecular regulations and biological processes may exist in different subtypes. By performing gene ontology enrichment analyses in the biological process category for the differentially expressed genes in subtypes, we found most of the over-expressed genes enriched in the processes related to inflammatory response, extracellular organization, cell adhesion functional terms, etc. It has been demonstrated that chronic inflammation is generally related to cancer progression [42,43,44], so these over-expressed genes may play important roles in tumor occurrence. In addition, we found that most of the under-expressed were genes for transcription-related functions, such as transcription and DNA-templated, negative regulation of transcription, and regulation of cell cycle, etc. This may imply that the difference of transcription regulations in different cancer subtypes may affect the response of temozolomide treatment.

In conclusion, we proposed SRF, an effective and flexible approach, to identify subtypes by integrating multi-omics data in cancer. Comprehensive experiments on multiple cancer datasets demonstrated that SRF could identify more clinically meaningful subtypes than most existing methods. SRF considers not only the similarity biases between samples, but also the weight of each data view in data integration. This information tends to enhance the accuracy of similarity estimation in data integration. In summary, our SRF approach provides an effective and simple tool to identify cancer subtypes by integrating multi-omics data. Although in this study we only integrated three types of omics data, SRF is an open integrative model and can easily be extended to integrate more omics data. We plan to integrate more omics data to identify subtypes more accurately by using SRF in our future work.

## Figures and Tables

**Table 1:** Statistics of datasets for four cancer types used in this study.

Datasets	Gene Expression (Features)	DNA Methylation (Features)	miRNA (Features)	Patients
BIC	17,814	23,094	354	105
GBM	12,042	1,305	534	215
LSCC	12,042	23,074	352	106
COAD	17,814	23,088	312	92

BIC: Breast invasive carcinoma; GBM: Glioblastoma multiforme; LSCC: Lung squamous cell carcinoma; COAD: Colon adenocarcinoma; microRNA: miRNA.

**Table 2:** The Cox log rank-test *p*-values in survival analyses for the identified subtypes by using different predicted similarity information in samples.

Datasets (# Subtypes)	Gene Expression	DNA Methylation	miRNA Expression	Integrative Data (SRF)
BIC (5)	1.59 × 10^−2^ (0.19)	3.67 × 10^−1^ (0.17)	3.56 × 10^−2^ (0.19)	1.20 × 10^−6^ (0.15)
GBM (3)	2.49 × 10^−3^ (0.20)	5.71 × 10^−3^ (0.22)	1.50 × 10^−3^ (0.23)	2.66 × 10^−4^ (0.21)
LSCC (4)	2.13 × 10^−2^ (0.26)	5.62 × 10^−1^ (0.19)	4.64 × 10^−3^ (0.25)	1.98 × 10^−3^ (0.26)
COAD (3)	6.64 × 10^−3^ (0.27)	3.84 × 10^−1^ (0.26)	2.61 × 10^−2^ (0.30)	1.96 × 10^−3^ (0.26)

The silhouette scores of clustering are denoted in parentheses.

**Table 3:** Comparison with six existing integrative methods.

Datasets (#Subtypes)	CNMF	iCluster	SNF	CC	SNF-CC	ANF	SRF
BIC (5)	9.74 × 10^−2^	2.04 × 10^−2^	1.35 × 10^−3^	2.81 × 10^−5^	2.94 × 10^−4^	7.60 × 10^−4^	1.20 × 10^−6^
GBM (3)	3.65 × 10^−1^	2.68 × 10^−3^	3.87 × 10^−4^	7.49 × 10^−1^	7.93 × 10^−4^	1.75 × 10^−2^	2.66 × 10^−4^
LSCC (4)	2.33 × 10^−2^	7.62 × 10^−3^	1.78 × 10^−2^	1.03 × 10^−2^	1.64 × 10^−2^	1.83 × 10^−2^	1.98 × 10^−3^
COAD (3)	2.22 × 10^−2^	2.46 × 10^−3^	3.60 × 10^−2^	3.72 × 10^−2^	3.80 × 10^−2^	3.92 × 10^−2^	1.96 × 10^−3^

Cox log-rank test *p*-values of the identified subtypes in survival analyses are evaluated. CNMF: Consensus non-negative matrix factorization; SNF: Similarity network fusion; CC: Consensus clustering; SNF-CC: Similarity network fusion- consensus clustering; ANF: Affinity network fusion; SRF: Similarity regression fusion.

**Table 4:** The average initial diagnosis ages and survival times of patients in the identified subtype clusters in BIC cancer datasets.

Subtype ID	C1 (*N* = 22)	C2 (*N* = 15)	C3 (*N* = 18)	C4 (*N* = 22)	C5 (*N* = 28)
Average age (years)	55.4	62.7	56.1	50.8	57.8
Average survival time (days)	1885.7	1116.7	3299.3	1623.5	2439.3

**Table 5:** The overlaps of the identified subtype clusters with PAM50 subtypes in BIC cancer datasets.

Subtype ID	C1 (*N* = 22)	C2 (*N* = 15)	C3 (*N* = 18)	C4 (*N* = 22)	C5 (*N* = 28)
Basal (23)	0 (0.0%)	1 (4.3%)	0 (0.0%)	18 (78.3%)	4 (17.4%)
Her2 (11)	0 (0.0%)	2 (18.2%)	0 (0.0%)	3 (27.3%)	6 (54.5%)
LumA (55)	17 (30.9%)	6 (10.9%)	16 (29.1%)	0 (0.0%)	16 (29.1%)
LumB (12)	4 (33.3%)	6 (50.0%)	0 (0.0%)	0 (0.0%)	2 (16.6%)
Normal (2)	0 (0.0%)	0 (0.0%)	1 (50.0%)	1 (50.0%)	0 (0.0%)
ER+ (80)	22 (27.5%)	13 (16.25%)	18 (22.5%)	5 (6.25%)	22 (27.5%)
ER− (24)	0 (0.0%)	1 (4.2%)	0 (0.0%)	17 (70.8%)	6 (25.0%)
PR+ (71)	19 (26.8%)	11 (15.5%)	18 (25.3%)	3 (4.2%)	20 (28.2%)
PR− (34)	3 (8.8%)	4 (11.8%)	0 (0.0%)	19 (55.9%)	8 (23.5%)

The estrogen receptor (ER) positive/negative and progesterone receptor (PR) positive/negative characteristics were examined.

**Table 6:** The average initial diagnosis ages and survival times of patients in the identified subtype clusters in GBM cancer datasets.

Subtype ID	C1 (*N* = 42)	C2 (*N* = 112)	C3 (*N* = 61)
Patients (Male:Female)	(24:18)	(69:43)	(41:20)
Average age (years)	46.4	58.8	54.8
Average survival time (days)	931.9	402.5	564.9

**Table 7:** The overlaps of the identified subtype clusters with the TCGA clinical subtypes in GBM cancer datasets.

Subtype ID	C1 (*N* = 42)	C2 (*N* = 112)	C3 (*N* = 61)
Classical (58)	2 (3.4%)	44 (75.9%)	12 (20.7%)
Mesenchymal (66)	0 (0.0%)	28 (42.4%)	38 (57.6%)
Neural (34)	3 (8.8%)	25 (73.5%)	6 (17.7%)
Proneural (57)	37 (64.9%)	15 (26.3%)	5 (8.8%)

## Supplementary Materials

The following are available online at http://www.mdpi.com/2073-4425/9/7/314/s1, Figure S1: The identified subtypes in LSCC cancer datasets, Figure S2: The identified subtypes in COAD cancer datasets, Table S1: GO enrichment analysis of differentially expressed genes between subtypes 1 and 2 in GBM cancer datasets, Table S2: GO enrichment analysis of differentially expressed genes between subtypes 1 and 3 in GBM cancer datasets.
