# Supplementary material for: A Similarity Regression Fusion Model for Integrating Multi-Omics Data to Identify Cancer Subtypes
Source: Genes (Basel). 2018 Jun 21;9(7):314. doi: 10.3390/genes9070314 (PMC6070922; doi:10.3390/genes9070314)
Supplement: Supplementary file 1 [file genes-09-00314-s001.pdf]

Supplementary Materials:

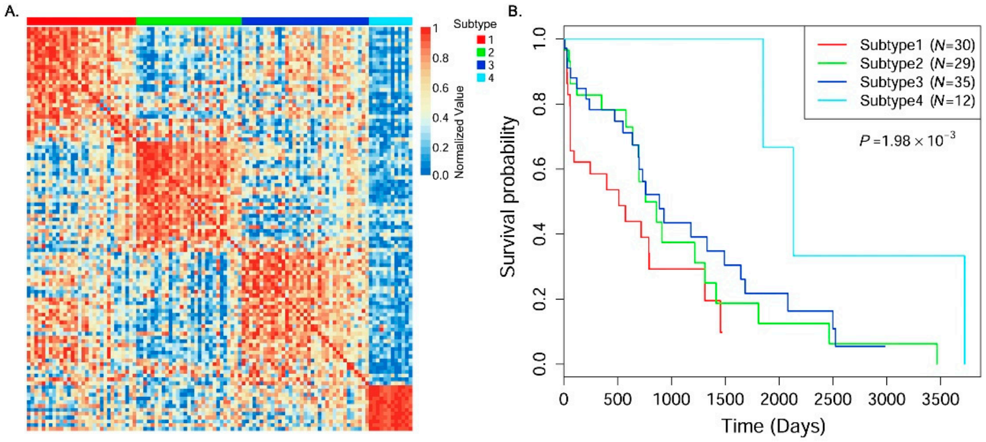

Figure S1. The identified subtypes in LSCC cancer datasets. (**A**) Heatmap of the predicted integrative similarity matrix between samples (row normalized, arranging samples by predicted subtype labels); (**B**) Kaplan–Meier survival probability curves of patients in the identified subtype clusters. The Cox log-rank test *p*-value was included.

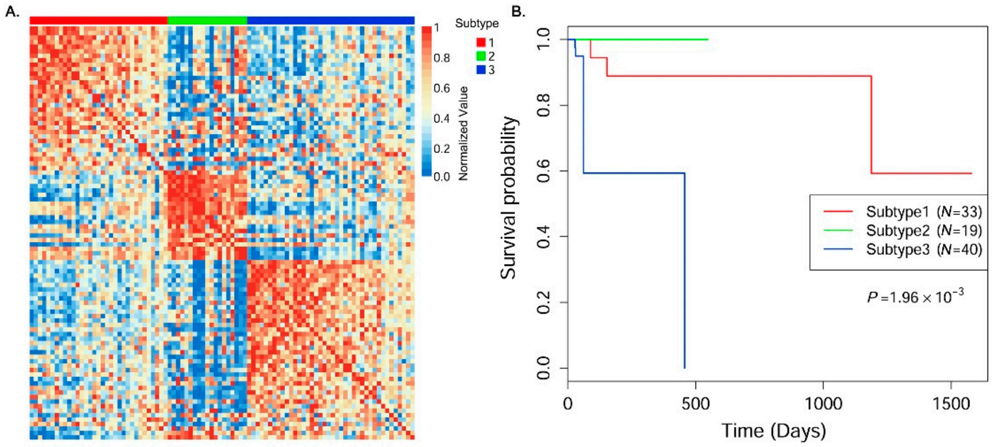

Figure S2. The identified subtypes in COAD cancer datasets. (**A**) Heatmap of the predicted integrative similarity matrix between samples (row normalized, arranging samples by predicted subtype labels); (**B**) Kaplan–Meier survival probability curves of patients in the identified subtype clusters. The Cox log-rank test *p*-value was included.

Table S1. Top 10 enriched GO terms (BH-FDR<0.01) in the GO biological process category of differentially expressed genes between subtypes 1 and 2 (gene expression in subtype 1 as baseline) in GBM cancer datasets.

**Subtype 1 vs. Subtype 2**	
GO enrichment of over-expressed genes	GO enrichment of under-expressed genes
1. Inflammatory response	1. Transcription, DNA-templated
2. Extracellular matrix organization	2. Negative regulation of transcription from RNA polymerase II promoter
3. Cell adhesion	3. Negative regulation of transcription, DNA-templated
4. Interferon-gamma-mediated signaling Pathway	4. Nervous system development
5. Angiogenesis	5. Positive regulation of transcription from RNA polymerase II promoter
6. Leukocyte migration	6. Transcription from RNA polymerase II Promoter
7. Signal transduction	7. Regulation of cell cycle
8. Aging	8. Regulation of transcription, DNA-templated
9. Platelet degranulation	9. Chromatin remodeling
10. Cellular response to lipopolysaccharide	10. G2/M transition of mitotic cell cycle

Table S2. Top 10 enriched GO terms (BH-FDR<0.0001) in the GO biological process category of differentially expressed genes between subtypes 1 and 3 (gene expression in subtype 1 as baseline) in GBM cancer datasets.

**Subtype 1 vs. Subtype 3**	
GO enrichment of over-expressed genes	GO enrichment of under-expressed genes
1. Inflammatory response	1. Transcription, DNA-templated
2. Immune response	2. Regulation of transcription, DNA-templated
3. Response to lipopolysaccharide	3. Covalent chromatin modification
4. Extracellular matrix organization	4. mRNA splicing, via spliceosome
5. Leukocyte migration	5. Negative regulation of transcription from RNA polymerase II promoter
6. Cell adhesion	6. Negative regulation of transcription, DNA-templated
7. Signal transduction	7. Positive regulation of transcription from RNA polymerase II promoter
8. Innate immune response	8. Chromatin remodeling
9. Chemotaxis	9. Protein sumoylation
10. Neutrophil chemotaxis	10. mRNA processing
